# Beta-Blocker-Related Atrioventricular Conduction Disorders—A Single Tertiary Referral Center Experience

**DOI:** 10.3390/medicina58020320

**Published:** 2022-02-20

**Authors:** Dragoș Traian Marius Marcu, Cristina Andreea Adam, Dan-Mihai Dorobanțu, Delia Lidia Șalaru, Radu Andy Sascău, Mircea Ovanez Balasanian, Liviu Macovei, Cătălina Arsenescu-Georgescu, Cristian Stătescu

**Affiliations:** 1 Department of Internal Medicine, University of Medicine and Pharmacy “Grigore T. Popa” Iași, 700115 Iasi, Romania; dragos.marcu11@yahoo.com (D.T.M.M.); deliasalaru@gmail.com (D.L.Ș.); radu.sascau@gmail.com (R.A.S.); ovanes718@yahoo.com (M.O.B.); liviughemacovei@yahoo.com (L.M.); catalina.arsenescu@gmail.com (C.A.-G.); cstatescu@gmail.com (C.S.); 2 Institute of Cardiovascular Diseases “Prof. Dr. George I. M. Georgescu” Iași, 700115 Iasi, Romania; 3 Children’s Health and Exercise Research Centre (CHERC), University of Exeter, Exeter EX1 2LU, UK; dd389@exeter.ac.uk; 4 Congenital Heart Unit, Bristol Royal Hospital for Children and Heart Institute, Bristol BS2 8BJ, UK

**Keywords:** beta-blocker, conduction disorder, adverse drug reactions, bradyarrhythmia, risk factor

## Abstract

*Background and Objectives*: Drug-related bradyarrhythmia is a well-documented major adverse event among beta-blocker users and a potential cause for hospitalization or additional interventions. Whether beta-blocker use is associated with specific bradyarrhythmia presentations, and how this relates to other predisposing factors, is not well known. We aim to evaluate the association between beta-blocker use and the type of atrioventricular (AV) conduction disorder in patients with symptomatic bradycardia. *Materials and Methods*: We conducted a retrospective cohort study on 596 patients with a primary diagnosis of symptomatic bradyarrhythmia admitted to a single tertiary referral center. Of the cases analyzed, 253 patients were on beta-blocker treatment at presentation and 343 had no bradycardic treatment. We analyzed demographics, clinical and paraclinical parameters in relation to the identified AV conduction disorder. A multivariate regression analysis was performed to explore factors associated with beta-blocker use. *Results*: Of the 596 patients (mean age 73.9 ± 8.8 years, 49.2% male), 261 (43.8%) had a third-degree AV block, 92 (15.4%) had a second-degree AV block, 128 (21.5%) had slow atrial fibrillation, 93 (15.6%) had sick sinus syndrome and 21 (3.5%) had sinus bradycardia/sinus pauses. Beta-blocker use was associated with the female gender (*p* < 0.001), emergency admission (*p* < 0.001), dilated cardiomyopathy (*p* = 0.003), the lower left ventricular ejection fraction (*p* = 0.02), mitral stenosis (*p* = 0.009), chronic kidney disease (*p* = 0.02), higher potassium levels (*p* = 0.04) and QRS duration > 120 ms (*p* = 0.02). Slow atrial fibrillation (OR = 4.2, *p* < 0.001), sick sinus syndrome (OR = 2.8, *p* = 0.001) and sinus bradycardia/pauses (OR = 32.9, *p* < 0.001) were more likely to be associated with beta-blocker use compared to the most common presentation (third-degree AV block), after adjusting for other patient characteristics. *Conclusions*: Beta-blocker use is more likely to be associated with slow atrial fibrillation, sick sinus syndrome and sinus bradycardia/pauses, compared to a second- or third-degree AV block, after adjusting for other patient factors such as gender, admission type, ECG, comorbidities, cardiac function and lab testing.

## 1. Introduction

Bradyarrhythmia is caused by intrinsic or extrinsic factors that lead to a dysfunction in the cardiac conduction system, with the most common extrinsic cause being iatrogenic, through routinely prescribed medication. Beta-adrenergic blockers, nondihydropyridine calcium channel blockers, digoxin, amiodarone or sympathomimetic antihypertensive agents such as clonidine are common agents that may lead to bradycardia [1,2,3].

Adverse drug reactions account for 6% of all annual hospitalizations and 2% of all deaths, causing losses of more than 650 million EURO (over 700 million USD to the health system). A systematic review of studies in the field indicates cardiovascular medication as the most frequent cause [4,5]. Moreover, according to the latest statistics, metoprolol as a beta-blocker was the fifth-most prescribed drug in the USA, with beta-blocker medication representing the second-most common class of drugs associated with the risk of hospitalization [6,7].

Beta-blockers are class II antiarrhythmic drugs mostly affecting the sinoatrial and atrioventricular nodes and are the most common cardiac medication associated with bradyarrhythmia, owing to its ubiquitous use in the treatment of cardiac disease. Studies on this topic clearly showed a higher incidence of bradyarrhythmia in patients treated with beta-blockers vs. placebo [8]. Most of the data on bradyarrhythmia associated with beta-blocker use come from heart failure trials. However, the data provided are incomplete, with bradycardia being, in most cases, an exclusion criterion. Factors associated with bradyarrhythmia and beta-blocker use are not well known, especially with the type of atrioventricular (AV) conduction disorder at presentation. Further research on this topic is necessary to identify potential risk factors that can predict the occurrence and type of symptomatic bradyarrhythmia [9,10,11]. 

In our study, we assessed a group of patients with symptomatic bradycardia with or without beta-blocker treatment, evaluating the relationship between treatment and the type of conduction disorder at presentation in relation to other patient factors. A better understanding of the clinical presentation of beta-blocker treatment-related bradyarrhythmia could improve the diagnosis accuracy of extrinsic vs. intrinsic AV conduction disorders and guide the indication for intervention. 

## 2. Materials and Methods

### 2.1. Study Design

We conducted a retrospective cohort study on 596 patients admitted to a single tertiary referral center—serving a population of approximately 5 million—in Northeast Romania, between December 2014 and January 2017 with a primary diagnosis of symptomatic bradyarrhythmia. We included patients admitted to our service with one of the following electrocardiographic diagnoses (based on a standard 12-lead electrocardiogram (ECG)): Sinus bradycardia, defined as a ventricular rate less than 60 beats per minute (BPM); sinus exit block, defined as a conduction failure from the sinus node to the atrium; second-degree AV block (Mobitz type I), defined as a progressive increase in PR interval duration, until a dropped *p* wave occurs; second-degree AV block (Mobitz type II), defined as intermittent dropped *p* waves with constant PR intervals; third-degree AV block, defined as bradycardia and complete dissociation of atrial and ventricular electrical conduction; slow atrial fibrillation, defined as atrial fibrillation with a heart rate of less than 60 BPM; sinus pauses, defined as no *p* wave for more than 0.2 s, on a sinus rhythm ECG; sick sinus syndrome, which is defined as alternating bradycardia–tachycardia and abnormalities that could not be classified in one of the previous variants. All included patients had symptoms such as syncope, dyspnea, dizziness, extreme fatigue or hemodynamic instability requiring hospitalization. 

Exclusion criteria were: <18 years of age; the conduction disorder could be attributed to an acute coronary syndrome; non-beta-blocker bradycardic treatment; recent cardiovascular surgery or interventional cardiology procedures; implantable device compatible with the pacing function; incomplete data regarding treatment or investigations of interest (e.g., unknown medication, echocardiographic data missing). 

The 596 patients enrolled in this study were divided into two groups based on beta-blocker use: BB+, on beta-blocker medication (*n* = 253), and BB−, without any type of bradycardic medication (*n* = 343). Socio-demographic characteristics, personal medical history, tobacco and alcohol consumption habits and chronic medication were obtained from the observation charts. Arterial hypertension and heart failure [12,13], renal dysfunction [14,15] and diabetes [16], were defined as per current guidelines. Laboratory results (complete blood count, erythrocyte sedimentation rate, serum creatinine, serum ionogram, lipid profile, liver enzymes (alanine aminotransferase, aspartate aminotransferase, gamma glutamyl transpeptidase), serum glucose, serum uric acid and international normalized ratio) were evaluated for each patient, with all results being expressed according to the International System of Units. ECG and transthoracic echocardiographic parameters were assessed. Body mass index (BMI) was calculated as weight (kg)/height (m^2^).

### 2.2. Statistical Analysis

Data were reported as the mean ± SD and as a number (frequency or percentages). Continuous variables were compared using the *t*-test (parametric analysis). Categorical variables were compared using the Fisher Exact test. 

To explore factors associated with beta-blocker use, a multivariable analysis was performed, using a backward stepwise regression approach: All parameters with a *p* < 0.1 in univariable logistic regression were included in the full (saturated) model, and at each step, the variable with the highest *p*-value (least model fit) was eliminated, until all independent predictors were left in the final (reported) model. At each step, the log-likelihood value was assessed to check changes in the model’s goodness of fit. Candidate variables and univariable regression results are detailed in the Supplementary material. 

A *p*-value of ≤0.05 was considered statistically significant. The descriptive analysis was performed using SPSS statistics software (version 20, IBM Corp. Armonk, NY, USA) and the univariable and multivariable analysis using STATA 16 SE (Stata Corp, College Station, TX, USA).

### 2.3. Ethics 

This study was approved by the Ethics Committee of the University of Medicine and Pharmacy “Grigore T. Popa” Iași and the Institute of Cardiovascular Diseases “Prof. Dr. George I.M. Georgescu”, Iași, and was conducted according to the Helsinki Declaration. All patients signed an informed consent statement on admission (Research Ethics Committee Notice dated 21.09.2019). 

## 3. Results

We analyzed a total of 596 patients with symptomatic atrioventricular conduction disorders, with a mean age of 73.9 (±8.8) years. The diagnoses identified were 19 (3.2%) cases of sinus disorders (bradycardia or pauses), 93 (15.6%) cases of sick sinus syndrome, 128 (21.5%) cases of slow atrial fibrillation, 261 (43.8%) cases of third-degree atrioventricular block, 92 (15.4%) cases of second-degree atrioventricular block and one (0.2%) case of first-degree atrioventricular block. Of these, 253 patients were under beta-blocker treatment and 343 had no bradycardic medication.

### 3.1. Demographics and Comorbidities

The demographic characteristics and comorbidities are presented in Table 1. The mean age was similar between BB+ and BB− groups, with more females in the BB+ group. Hypertension (70.4% vs. 68.5%, *p* = 0.65) and diabetes mellitus (24.5% vs. 19.8%, *p* = 0.19) were more frequent in the BB+ group. Moreover, more patients in this group presented with associated acute kidney injury (*p* = 0.001), chronic kidney disease regardless of exacerbation (*p* = 0.001) and heart failure (*p* < 0.001).

### 3.2. Clinical, Echocardiographic and Biological Patient Characteristics

#### 3.2.1. Clinical and ECG Findings

Table 2 shows the admission vitals and the electrocardiographic findings. Emergency hospitalization (56.5% vs. 39.7%, *p* < 0.001) and temporary cardiac pacing (17.4% vs. 11.4%, *p* = 0.04) were more frequent in the BB+ group. The mean values and SD of systolic blood pressure (*p* = 0.04) and diastolic blood pressure (*p* = 0.63) were slightly increased in the BB- group, as opposed to the higher mean values of heart rate (*p* = 0.002) in patients with beta-blocker use. The second-degree AV block (*p* < 0.001) was more frequent in the BB- group, while slow ventricular response atrial fibrillation (*p* = 0.007), sinus pauses and sinus bradycardia (*p* < 0.001) were diagnosed more in the BB+ group. The left bundle branch block (LBBB) morphology (*p* = 0.08) during conduction disorder was more frequent in the BB+ group.

#### 3.2.2. Laboratory Tests

Regarding the assessed blood tests, lower levels of serum natrium, a lower estimated glomerular filtration rate (eGFR) and higher levels of serum potassium were found in the BB+ group. The results of the laboratory tests analysis are detailed in Supplementary Table S1.

#### 3.2.3. Echocardiography

The echocardiographic findings (Table 3) showed that left ventricular dilation (*p* = 0.002), mitral stenosis (*p* = 0.002) and biatrial enlargement (*p* = 0.001) were more frequent in the BB+ group. The mean value of the left ventricular ejection fraction (*p* < 0.001) was higher in the BB− group.

### 3.3. Patient Factors Associated with Beta-Blocker Use

Slow atrial fibrillation (OR = 4.2, *p* < 0.0001), sick sinus syndrome (OR = 2.8, *p* = 0.001) and sinus bradycardia/pauses (OR = 32.9, *p* < 0.0001) were more likely to be associated with beta-blocker use compared to the most common presentation (third-degree AV block), but second-degree AV block was not (OR = 0.84, *p* = 0.56), after adjusting for patient demographic, clinical and biological characteristics. Beta-blocker use was also associated with the female gender (*p* < 0.0001), emergency admission (*p* < 0.0001), dilated cardiomyopathy (*p* = 0.003), lower left ventricular ejection fraction (*p* = 0.02), mitral stenosis (*p* = 0.009), chronic kidney disease (*p* = 0.02), higher potassium levels (*p* = 0.04) and QRS duration > 120 ms (*p* = 0.02). The results of the univariable analysis are detailed in Supplementary Table S2 (other analyzed variables were age, atrial flutter, left anterior fascicular block, left posterior fascicular block, diastolic blood pressure, arterial hypertension grade, diabetes mellitus, aortic stenosis, aortic regurgitation, pulmonary regurgitation, tricuspid regurgitation, left ventricular hypertrophy, transaminases, total cholesterol, high-density lipoprotein cholesterol, low-density lipoprotein cholesterol, triglycerides, lactate dehydrogenase and creatine kinase-MB), and those of the multivariable analysis in Table 4. 

### 3.4. Beta-Blocker Agents

Most patients were treated with carvedilol (35.2%), followed by bisoprolol (30.8%) and metoprolol (14.2%). Details are shown in Table 5.

## 4. Discussion

Our results indicate that beta-blocker use is more likely to be associated with slow atrial fibrillation, sinus bradycardia/pauses and sick sinus syndrome than severe types of AV block (second- and third-degree AV block). These findings were consistent after adjusting for other factors, and in line with other data in the literature [17]. Thus, the data provided by our study are of both therapeutic and prognostic importance and complement existing data on atrioventricular conduction disorders related to the use of bradycardic medication. Considering both our findings and literature data, second and third-degree AV blocks tend to be independent of beta-blocker use. Consequently, less-severe bradyarrhythmia or slow atrial fibrillation tend to be associated with beta-blocker medication and, most likely, a higher chance of reversibility.

Currently, there is controversy regarding the management of possibly drug-induced bradyarrhythmia. According to current guidelines, pacemaker implantation (PM) is not necessary for bradyarrhythmia considered to be drug induced. However, studies in the field showed that about 85% of patients suspected of iatrogenic bradycardia due to medication will require permanent pacemaker implantation. This conclusion follows the observation that most AV blocks cannot be attributed to medication alone, with drugs being a mere trigger of an underlying condition. In addition, delay in PM implantation may result in adverse effects on both the patient’s quality of life and long-term prognosis as well as the financial burden on the healthcare system resulting from repeated and prolonged hospitalizations [1,18]. Considering these effects, a paradigm shift appears to be necessary, and there are already recommendations for the implantation of a PM despite bradycardic treatment, under certain conditions [19]. There are opinions suggesting that for an elderly patient with an AV block requiring hospitalization and temporary pacing in an emergency setting, long-term management will always require the implantation of a PM, with any delays in this regard being unnecessary [20]. Several studies have shown that pacemaker implantation may be beneficial in patients with a history of unexplained syncope and criteria for sinus node or atrioventricular node dysfunction upon electrophysiological study. In octogenarian patients, this approach is likely to increase syncope-free survival and decrease mortality [21,22,23].

Our results support the decision to use a pacemaker in an AV block (second- or third- degree), even when a beta-blocker is used, and carefully evaluate potential reversibility when other types of bradycardias are observed, especially when additional risk factors for reversible causes are present.

From a prognostic point of view, current studies indicate age, the female gender and associated comorbidities as risk factors for medication-related bradyarrhythmia [24,25]. By analyzing the demographic parameters collected, we observed that age did not register significant differences between the two groups. Instead, the female gender was more prevalent in the BB+ group, with a statistically significant *p*-value, maintaining its association after performing multivariate regression. Moreover, the female gender is also considered a general risk factor for ADRs, but particularly for those associated with cardiological and psychiatric medication. Underlying these predispositions, there are several yet understudied peculiarities related to metabolism, hormonal balance and medication management [26,27].

The need for emergency hospitalization and temporary pacing was significantly higher in the BB+ group, which may be associated, on one hand, with the presence of important comorbidities (heart failure, renal dysfunction) that contributed to the patient’s hemodynamic instability, and on the other hand, with pre-existing damage to the excitoconductive system, suggested by the presence of prior LBBB. At the same time, the need for emergency temporary pacing can be explained by the increased refractoriness of the myocardium under the effect of medication and the tendency for a lower block level. Research on ADRs points to cardiovascular medication and, by default, beta-blocker medication as the most frequent agents underlying the need for emergency hospitalization [28,29,30].

Furthermore, heart failure, regardless of NYHA functional class, was more frequent in the BB+ group. This can be attributed to the extensive use of beta-blocker medication in heart-failure patients in terms of guideline recommendations and their beneficial effects [12,19,31]. A meta-analysis of adverse drug effects in patients with heart failure showed that beta-blocker medication had a risk of 1 in 26 patients per year of developing bradycardia. Moreover, the risk of discontinuation of medication due to bradycardia was significantly higher than placebo. It should be noted, however, that despite these adverse effects, the benefits of treatment for these patients far outweigh the drawbacks of therapy [32]. Another review of available studies mainly targeting the female population with heart failure highlighted their increased risk of developing general adverse reactions to beta-blockers and the need for further analysis of this category of patients [33]. Of note is the category of patients with heart failure and atrial fibrillation, in whom beta-blocker treatment with the maintenance of a target heart rate below 75 bpm was associated with a significantly higher rate of symptomatic bradyarrhythmia, but without achieving the expected beneficial effect in the treatment of heart failure [34]. After multivariate analysis, LVEF and left ventricular dilatation, as parameters associated with heart failure, maintained their predictive power. 

Renal dysfunction had a strong statistical significance in the BB+ group, both in terms of acute renal dysfunction and chronic kidney disease. It should be emphasized that chronic kidney disease showed a statistically significant association with the BB+ group even in the absence of exacerbation, which highlights the role of related chemo-metabolic changes in creating a predisposition towards the development of atrio-ventricular conduction disorders associated with beta-blocker consumption. This is consistent with literature data on the frequency of ADR inversely proportional to eGFR. The high prevalence of beta-blockers with predominantly hepatic metabolism (metoprolol, nebivolol) can be explained in the context of associated comorbidities and possibly in the context of drug–drug interactions [35,36,37]. Ion balance shows significantly lower sodium and significantly higher potassium levels in patients on beta-blocker treatment. These data are most likely related to the comorbidities and lower eGFR values in the BB+ group. Although low, it is also worth mentioning the possibility of increased serum potassium levels due to beta-blocker treatment, which may potentiate the bradycardic effect of medication [38,39,40].

In terms of the beta-blocking agent used, most cases fell into one of four categories: Patients treated with bisoprolol, patients treated with carvedilol, patients treated with nebivolol and patients treated with metoprolol. This is consistent with the frequency of heart-failure cases in the BB+ group, with the agents mentioned above part of the first-line treatment of chronic heart failure according to current guidelines [12,31]. Of these, two agents, bisoprolol and carvedilol, were found in more than half of the cases, with carvedilol being the most used. It is worth mentioning that compared to carvedilol, bisoprolol and metoprolol are associated with a greater decrease in heart rate and a higher risk of bradyarrhythmia [41,42]. It should also be noted that the average dose for each agent was significantly lower than the maximum therapeutic dose [43,44,45]. The large clinical trials considered the maximum therapeutic doses that Carvedilol to be 25 mg twice daily, Metoprolol to be 200 mg daily, Bisoprolol to be 10 mg daily and Nebivolol to be 10 mg daily, although clinical reality has shown that the mean daily dose is usually less than 50% of these targets, which is also seen in our results [46]. This may be related to the prevalence of comorbidities in the BB+ group, which may have imposed therapeutic limitations.

## 5. Limitations

Our study presents several limitations due to its retrospective nature, the number of cases analyzed and the specificity of our center. Moreover, the adverse effects of beta-blocker medication are a long-studied topic in the literature, with information dating back to the early use of this type of medication. This has been a challenge in the development of our topic, and highlighting a relatively unexplored aspect, our study is nevertheless one of the few recent studies in the field. Since data were extracted from hospital records, many cases being emergency admissions, some were incomplete. We decided to exclude those records where critical information was missing, or unreliable. This was in order to minimize the risk of misclassification, introducing a limited risk of selection bias, as factors leading to incomplete data seemed to be random. This allowed for the accurate classification of outcomes. More so, there was a good mix of patients, belonging to both clinical and emergency/elective status. However, several parameters, including the type of beta-blocker medication and dose administered, are susceptible to recall bias. Considering the underrepresentation of certain beta-blocking agents scarcely used in our region, we cannot exclude the possibility of different results in the case of the inclusion of beta-blockers with different properties.

## 6. Conclusions

Our results indicate that beta-blocker use is more likely to be associated with slow atrial fibrillation, sick sinus syndrome and sinus bradycardia or sinus pauses, compared to third- or second-degree AV block, after adjusting for other patient factors such as gender, admission type, ECG, comorbidities, cardiac function and lab testing. These findings are of dual importance, both in managing the risk of developing symptomatic bradyarrhythmia under beta-blocker medication and in subsequent management. Depending on the type of atrioventricular conduction disorder, the subsequent decision of permanent electrical pacing or a simple adjustment of medication doses becomes easier to make.

## Figures and Tables

**Table 1:** Demographics and comorbidities.

Parameters	BB+ (*n* = 253)	BB− (*n* = 343)	Total (*n* = 596)	*p* Value
Age (yrs.)	73.79 ± 8.57	74.00 ± 8.97	73.9 ± 8.8	0.77
Females	150 (59.3%)	153 (44.6%)	303 (50.6%)	<0.001
Originating area (urban vs. rural)	132 (52.2%)	161 (46.9%)	293 (49.2%)	0.21
Body mass index, kg/m^2^	27.10 ± 4.54	27.19 ± 4.45	27.1 ± 4.6	0.81
Hypertension	175 (70.4%)	235 (68.5%)	413 (69.3%)	0.65
Diabetes mellitus	62 (24.5%)	68 (19.8%)	130 (21.8%)	0.19
Acute kidney injury	46 (18.2%)	30 (8.7%)	76 (12.8%)	0.001
Chronic kidney disease	74 (29.2%)	60 (17.5%)	134 (22.5%)	0.001
Heart failure	93 (36.8%)	78 (22.7%)	171 (28.7%)	<0.001

**Table 2:** Clinical and ECG findings.

	BB+ (*n* = 253)	BB− (*n* = 343)	Total (*n* = 596)	*p* Value
Clinical presentation				
Emergency admission	143 (56.5%)	136 (39.7%)	279 (46.8%)	<0.001
SBP, mmHg	142.76 ± 27.31	147.17 ± 26.27	145.3 ± 26.8	0.04
DBP, mmHg	74.46 ± 13.84	74.99 ± 12.66	74.8 ± 13.2	0.63
HR, beat/min	58.10 ± 24.26	52.42 ± 19.62	54.83 ± 21.9	0.002
Temporary Cardiac Pacing	44 (17.4%)	39 (11.4%)	83 (13.9%)	0.04
Syncope	130 (51.4%)	140 (40.8%)	270 (45.3%)	0.01
Electrocardiogram				
First-degree AV block	1 (0.40%)	-	1 (0.2%)	0.42
Second-degree AV block	23 (9.1%)	69 (20.1%)	92 (15.4%)	<0.001
Third-degree AV block	105 (41.5%)	156 (45.5%)	261 (43.8%)	0.35
Slow atrial fibrillation	69 (27.3%)	61 (17.8%)	128 (21.5%)	0.007
Sick sinus syndrome	39 (15.4%)	54 (15.7%)	93 (15.6%)	0.9
Sinus bradycardia and sinus pauses	16 (6.4%)	3 (0.9%)	19 (3.2%)	<0.001
Heart rhythm prior to conduction disorder				
Sinus rhythm	159 (62.8%)	264 (77%)	423 (71.0%)	<0.001
Atrial fibrillation	93 (36.8%)	78 (22.7%)	171 (28.7%)	<0.001
Atrial flutter	1 (0.4%)	1 (0.3%)	2 (0.3%)	0.9
QRS morphology prior to conduction disorder				
LBBB	25 (9.9%)	20 (5.8%)	45 (7.6%)	0.08
RBBB	43 (17%)	49 (14.3%)	92 (15.4%)	0.42
Absence of bundle branch block	185 (73.1%)	274 (79.9%)	459 (77.0%)	0.06
QRS morphology during conduction disorder				
LAFB	42 (16.6%)	65 (19.0%)	107 (18.0%)	0.51
LPFB	3 (1.2%)	7 (2.0%)	10 (1.7%)	0.52
LBBB	37 (14.6%)	31 (9.0%)	68 (11.4%)	0.08
RBBB	64 (25.3%)	81 (23.6%)	145 (24.3%)	0.42
Absence of bundle branch block	152 (60.1%)	231 (67.3%)	479 (80.4%)	0.34

AV: Atrioventricular; SBP: Systolic blood pressure; DBP: Diastolic blood pressure; HR: Heart rate; LBBB: Left bundle branch block; RBBB: Right bundle branch block; LAFB: Left anterior fascicular block; LPFB: Left posterior fascicular block.

**Table 3:** Echocardiographic measurements.

Measurement	BB+ (*n* = 253)	BB− (*n* = 343)	Total (*n* = 596)	*p* Value
Left ventricular dilation	48 (19.0%)	34 (9.9%)	82 (13.8%)	0.002
Aortic stenosis	52 (20.6%)	71 (20.7%)	123 (20.6%)	0.9
Mitral stenosis	17 (6.7%)	6 (1.7%)	23 (3.9%)	0.002
Mitral annular calcification	153 (60.5%)	199 (58%)	352 (59.1%)	0.55
Biatrial Enlargement	183 (72.3%)	201 (58.6%)	384 (64.4%)	0.001
Normal systolic function	114 (45.1%)	177 (51.6%)	291 (48.8%)	0.11
Mild systolic dysfunction	93 (36.8%)	138 (40.2%)	231 (38.8%)	0.39
Moderate systolic dysfunction	27 (10.7%)	20 (5.8%)	47 (7.9%)	0.03
Severe systolic dysfunction	19 (7.5%)	8 (2.3%)	27 (4.5%)	0.04
Left ventricular septal hypertrophy	231 (91.3%)	310 (90.4%)	541 (90.8%)	0.77
Pulmonary hypertension probability	130 (51.4%)	144 (58%)	274 (46.0%)	0.28
Mitral regurgitation	200 (79.1%)	259 (75.5%)	459 (77.0%)	0.32
Aortic regurgitation	114 (45.1%)	159 (46.4%)	273 (45.8%)	0.80
Tricuspid regurgitation	180 (71.1%)	244 (71.1%)	424 (71.1%)	0.9
Pulmonary regurgitation	26 (10.3%)	34 (9.9%)	60 (10.1%)	0.89
Left ventricular ejection fraction, %	50.4 ± 12.3	53.8 ± 9.3	52.4 ± 50	<0.001
Interventricular septum, mm	12.5 ± 2.2	12.4 ± 1.9	12.4 ± 12	0.41

All values are expressed as n (%) or mean ± standard deviation (SD).

**Table 4:** Atrioventricular conduction disorders associated with beta-blocker use after adjusting for patient-dependent factors.

	Multivariable Analysis		
Parameter *	OR	*p* Value	95%CI
Third-degree AV block	Baseline	Baseline	Baseline
Second-degree AV block (Type 2)	0.84	0.560	0.46–1.5
Slow atrial fibrillation	4.2	<0.001	2.4–7.3
Sick sinus syndrome	2.8	0.001	1.5–5.1
Sinus bradycardia/sinus pauses	32.9	<0.001	8.4–128.8
Female gender	2.6	<0.001	1.7–3.9
Emergency admission	3.1	<0.001	2.0–4.7
QRS duration >120 ms	1.7	0.015	1.1–2.5
Chronic kidney disease	1.8	0.016	1.1–2.8
Left ventricular dilation	2.5	0.003	1.4–4.6
Mitral stenosis	4.2	0.009	1.4–12.4
Left ventricular ejection fraction	0.98	0.016	0.96–0.99
K	1.3	0.041	1.0–1.7
Glucose	1.005	0.015	1.00–1.01

AV: Atrioventricular; CI: Confidence interval; K: Potassium; OR: Odds ratio; * Only one case of second-degree AV block (type 1), not included in the model.

**Table 5:** Beta-blocker agents identified in the BB+ group.

Agent	Total BB+ (*n* = 253)	Dose (mg/day) Mean ± SD
Bisoprolol	78 (30.8%)	5.2 ± 3.3
Carvedilol	89 (35.2%)	9.3 ± 5.4
Nebivolol	35 (13.8%)	4.3 ± 1.6
Metoprolol	36 (14.2%)	69.4 ± 34.9
Atenolol	6 (2.4%)	50.0 ± 0.0
Betaxolol	3 (1.2%)	20.0 ± 0.0
Propranolol	1 (0.4%)	10.0
Sotalol	5 (2.0%)	96.0 ± 15.6

All values are expressed as n (%) or mean ± standard deviation (SD).

## Data Availability

The data that support the findings of this study are available on request from the corresponding author. The data are not publicly available due to privacy or ethical restrictions.

## Supplementary Materials

The following are available online at https://www.mdpi.com/article/10.3390/medicina58020320/s1, Table S1: Laboratory tests; Table S2: Candidate variables and univariable regression results.
