# Supplementary material for: Beta-Blocker-Related Atrioventricular Conduction Disorders—A Single Tertiary Referral Center Experience
Source: Medicina (Kaunas). 2022 Feb 20;58(2):320. doi: 10.3390/medicina58020320 (PMC8877089; doi:10.3390/medicina58020320)
Supplement: Supplementary file 1 [file medicina-58-00320-s001.zip › medicina-1534287-supplementary.pdf]

Table S1. Laboratory tests.

Test	BB+ (n=253)	BB- (n=343)	Total (n=596)	P value	Normal range and unit of measure
Na	137.6 ± 4.8	140 ± 3.4	138.4 ± 4.1	<0.001	135 – 148 mmol/L
K	4.6 ± 0.94	4.4 ± 0.59	4.5 ± 0.76	0.01	3.5 – 5.1 mmol/L
Ca	1.2 ± 0.09	1.2 ± 0.08	1.2 ± 0.08	0.06	1.16 – 1.35 mmol/L
Serum creatinine	1.3 ± 0.68	1.2 ± 0.76	1.2 ± 0.73	0.06	0.5 – 0.9 mg/dl
Urea	63.8 ± 36	56.2 ± 31.2	59.4 ± 33.5	0.01	10 -50 mg/dl
eGFR	55.5 ± 21.2	62.9 ± 20.2	59.7 ± 21	<0.001	>90 ml/min/1.73 m^2
Uric acid	6.9 ± 1.7	6.5 ± 1.7	6.7 ± 1.7	0.01	2.4-5.7 mg/dl
Glucose	129.1 ± 63.5	117.4 ± 36.2	122.3 ± 50	0.01	75 – 115 mg/dl
AST	44.7 ± 60.2	44.6 ± 91.2	44.7 ± 79.5	0.98	0 – 31 U/L
ALT	38.5 ± 42	43.1 ± 87.4	41.1 ± 71.7	0.43	0 – 31 U/L
GGT	64.3 ± 79.8	52.9 ± 60.6	57.7 ± 69.5	0.05	7 – 32 U/L
Cholesterol	182.7 ± 46.7	181.1 ± 46.1	181.8 ± 46.3	0.68	0-200 mg/dl
LDL-cholesterol	113.8 ± 37.8	110.6 ± 37.7	112 ± 37.8	0.31	0-160 mg/dl
HDL-cholesterol	46.5 ± 15	47.8 ± 13.6	47.2 ± 14.2	0.27	42-98 mg/dl
Triglyceride	118.6 ± 67	120.7 ± 81.9	119.8 ± 76	0.74	35-150 mg/dl
INR	1.5 ± 1.1	1.2 ± 0.79	1.4 ± 0.96	<0.001	0.76 – 1.24
CRP	23.2 ± 40.9	15.7 ± 27.5	18.98 ± 33.9	0.02	0-5 mg/l
WBC	9250.5 ± 4929.5	8515.2 ± 2982.9	8827.4 ± 3941.9	0.03	4000 – 10500/mm^3
RBC	4.4 ± 0.62	4.5 ± 0.6	4.5 ± 0.60	0.32	4.2 – 5.4 mil/mm^3
Hb	13.2 ± 1.7	13.4 ± 1.7	13.3 ± 1.7	0.09	12.5 – 16 g/dl
PCV	39.7 ± 5	40.6 ± 5.1	40.2 ± 5.1	0.02	37 – 47%
MCV	89.6 ± 5.6	90 ± 5.7	89.3 ± 5.7	0.33	78 – 100 fl
MCH	29.6 ± 2.4	29.7 ± 2.1	29.6 ± 2.2	0.45	32 – 36 g/dl
MCHC	32.8 ± 1	32.9 ± 1.1	32.9 ± 1.1	0.83	27-31 pg
Platelets	214919.4 ± 75624.608	220017.2 ± 74853.1	217853.2 ± 75160.5	0.41	150000-450000/mm^3

Na: sodium; K: potassium; Ca: calcium; AST: aspartate aminotransferase; ALT: alanine aminotransferase; GGT: gamma glutamyl transferase; LDL: low-density lipoprotein; HDL: high-density lipoprotein; INR: International Normalized Ratio; CRP: C-reactive protein; MCV: mean corpuscular volume; MCH: mean corpuscular hemoglobin; MCHC: mean corpuscular hemoglobin concentration.

Table S2. Candidate variables and univariable regression results.

	Univariate regression			Multivariate regression		
**Parameter**	**OR**	**95% CI**	**p value**	**OR**	**95%CI**	**p value**
**Bradyarrhythmia type**						
Third degree AV block baseline	Baseline	Baseline	Baseline	Baseline	Baseline	Baseline
Type 2 Second degree AV block	0.49	0.29-0.84	0.01	0.84	0.46-1.5	0.560
Slow atrial fibrillation	1.63	1.06-2.49	0.02	4.18	2.4-7.3	<0.001
Sick sinus syndrome	1.07	0.66-1.73	0.8	2.8	1.5-5.1	0.001
Sinus bradycardia + sinus pauses	8.91	2.56-31.02	0.001	32.9	8.4-128.8	<0.001
**Bundle branch block**						
None	Baseline	Baseline	Baseline			
RBBB	1.29	0.82-2.0	0.25			
LBBB	1.85	0.99-3.4	0.05			

Atrial fibrillation (non-slow)	1.97	1.4-2.8	<0.0001			
Females	1.8	1.3-2.51	<0.0001	2.6	1.7-3.9	<0.001
Emergency admission	1.97	1.42-2.75	<0.0001	3.1	2.0-4.7	<0.001
Systolic blood pressure	0.99/mmHg	0.98-0.99	0.05			
Syncope	1.5	1.1-2.1	0.01			
QRS duration >120 ms	1.38	0.98-1.93	0.06	1.7	1.1-2.5	0.015
Heart failure symptoms	2.0	1.4-3.8	<0.001			
Acute renal failure	2.31	1.4-3.8	0.001			
CKD without exacerbation	1.67	1.0-2.6	0.02			
CKD	1.94	1.32-2.87	0.001	1.8	1.1-2.8	0.016
Pulmonary hypertension	1.46	1.1-2.0	0.02			
Left ventricular dilation	2.12	1.32-3.41	0.002	2.5	1.4-4.6	0.003
Mitral stenosis	4.04	1.57-10.4	0.004	4.2	1.4-12.4	0.009
Severe mitral regurgitation	2.34	1.4-3.9	<0.001			
Left ventricular ejection fraction	0.97/%	0.95-0.98	<0.0001	0.98	0.96-0.99	0.016
**Left ventricular dysfunction**						
None	Baseline	Baseline	Baseline			
Mild left ventricular dysfunction	1.0	0.73-1.5	0.8			
Moderate left ventricular dysfunction	2.1	1.1-3.9	0.02			
Severe left ventricular dysfunction	3.7	1.6-8.7	0.003			
Na	0.92	0.88-0.96	<0.0001			
K	1.35	1.09-1.69	0.006	1.3	1.0-1.7	0.041
Ca	0.17	0.02-1.1	0.07			
Creatinine	1.2	0.97-1.6	0.08			
Urea	1.01	1.0-1.01	0.007			
eGFR	0.98	0.98-0.99	<0.0001			
Uric acid	1.1	1.0-1.3	0.01			
GGT	1.01	0.99-1.0	0.05			
Glucose	1.004	1.001-10.008	0.007	1.005	1.00-1.01	0.015
CRP	1.001	1.00-1.01	0.03			
Hemoglobin	0.92	0.84-1.0	0.09			
WBC	1.001	1.00-1.01	0.03			

AV: atrioventricular; CI: confidence interval; OR: odds ratio; RBBB: right bundle branch block; LBBB: left bundle branch block; CKD: chronic kidney disease; Na: sodium; K: potassium; Ca: calcium; eGFR: estimated glomerular filtration rate; GGT: gamma-glutamyl transferase; CRP: C-reactive protein; WBC: white blood cells;
